# Relating Information, Encoding and Adaptation: Decoding the Population Firing Rate in Visual Areas 17/18 in Response to a Stimulus Transition

**DOI:** 10.1371/journal.pone.0010327

**Published:** 2010-04-27

**Authors:** David Eriksson, Sonata Valentiniene, Stylianos Papaioannou

**Affiliations:** 1 Cortical Function and Dynamics, Max Planck Institute for Brain Research, Frankfurt, Germany; 2 Brain Research, Department of Neuroscience, Karolinska Institute, Stockholm, Sweden; Lund University, Sweden

## Abstract

Neurons in the primary visual cortex typically reach their highest firing rate after an abrupt image transition. Since the mutual information between the firing rate and the currently presented image is largest during this early firing period it is tempting to conclude this early firing encodes the current image. This view is, however, made more complicated by the fact that the response to the current image is dependent on the preceding image. Therefore we hypothesize that neurons encode a combination of current and previous images, and that the strength of the current image relative to the previous image changes over time. The temporal encoding is interesting, first, because neurons are, at different time points, sensitive to different features such as luminance, edges and textures; second, because the temporal evolution provides temporal constraints for deciphering the instantaneous population activity. To study the temporal evolution of the encoding we presented a sequence of 250 ms stimulus patterns during multiunit recordings in areas 17 and 18 of the anaesthetized ferret. Using a novel method we decoded the pattern given the instantaneous population-firing rate. Following a stimulus transition from stimulus A to B the decoded stimulus during the first 90ms was more correlated with the difference between A and B (B-A) than with B alone. After 90ms the decoded stimulus was more correlated with stimulus B than with B-A. Finally we related our results to information measures of previous (B) and current stimulus (A). Despite that the initial transient conveys the majority of the stimulus-related information; we show that it actually encodes a difference image which can be independent of the stimulus. Only later on, spikes gradually encode the stimulus more exclusively.

## Introduction

Our brain has to deal with many types of sudden changes in our visual surrounding, such as a tiger that appears behind a tree, or the quick eye movements of the person we are having a conversation with. The visual system responds vigorously to these transient stimuli. Experimentally this initial transient response has been evoked by everything from luminance changes [1]–[7] to natural vision movies [8], [9], and saccades [10]. In a classical study it was shown that the initial spikes conveys most information about the stimulus, whereas later spikes convey less information [11], [12]. Does this mean that the stimulus is most accurately represented during the first few spikes, and less during later spikes?

The first few spikes convey a large amount of information about the stimulus and therefore they also have a large signal to noise ratio. The signal tells what message is being encoded. A signal in this context is “good” if it corresponds to the currently presented stimulus, and “bad” if it corresponds to another stimulus that, for example, was presented 1 minute earlier. A large signal to noise ratio can be the result of a good signal, a low noise level, or a combination of both. The noise level is in general dependent on the absolute firing rate. The signal on the other hand does not have such a dependency. Therefore we wanted to examine what image is encoded in general and in particular whether the first few spikes encode the same image as the later spikes.

The latter question is especially interesting because neurons are sensitive to different types of features at different time points after image onset. In the macaque monkey neurons are sensitive to local features and edges at 40–60ms, surface borders at 60–80ms, interior of the surface 90–110ms, and three dimensional shape and attention after 110ms [13], [14]. Different aspects of the same image are therefore represented at different time points. In this paper we examine a complementary view. Does the encoded image change over time, e.g. can the orientation in the encoded image first be 0 degrees and then later be 90 degrees despite that the stimulus image is constant?

The represented image is likely to change over time even if the stimulus is constant. This is due to strong temporal modulations of the instantaneous firing rate [15], caused by ON and OFF responses, rebound bursts, oscillations and adaptation. Although it is impossible to describe the temporal modulation of the instantaneous firing rate using only one of these properties, adaptation is probably most relevant for this study. Adaptation takes the stimulation history as a reference for the representation of future stimuli. In a recent review many possible benefits of adaptation were discussed, but the conclusion was that the actual benefits remain unclear [16]. One open question is how adaptation affects population coding.

For the individual neuron adaptation typically causes high firing rates in response to a temporal change in the stimulus. In a neuronal population each neuron samples from a unique retinal position, which enables the encoding of temporal changes at every retinal position. Thus, the instantaneous population rate may encode a difference image rather than the current retinal image [10]. The difference image and current image may be independent from each other. This means that a pattern change not only delays stimulus related information [17], but may even make neurons convey information that contradicts the stimulus.

The only possibility for having a continuous representation of the retinal image is to take the temporal modulation of the instantaneous firing rate into account [18], [19]. This also means that the retinal image is only available for analysis (e.g. by the extra striate areas) if the temporal modulation is considered. In contrast, in most computational models and data analysis it is assumed that the retinal image is available directly in the instantaneous firing rate because the sensitivity to a certain image feature is defined in terms of the instantaneous firing rate [13], [14], [20]–[24]. Therefore we are interested in what image is encoded by the instantaneous firing rate.

If neurons encode the difference image initially, when do they switch to encode the current stimulus? Neurons decrease their dependency on the previous stimulus for up to 500 ms before the firing rate eventually becomes independent on the previous stimulus [7], [17], [25]–[27]. This history dependency can be due to for example adaptation, OFF-responses and rebound responses from previous stimulus [25], [28], [29]. Since the dependency on previous stimulus decreases over time the current stimulus may get more and more exclusively encoded. In contrast to this, information analysis studies have shown that information about the current stimulus decreases over time [11], [30]–[32].

To relate the decrease in information about the current stimulus to the increase in the exclusiveness of the current stimulus encoding, we have, for each time point after the stimulus transition, extracted the encoded image and compared that to the amount of information that the neurons convey about the previous and the current image. In general decoding quantifies which signal is encoded, whereas information-measures quantify how accurately that signal is encoded. Our results suggest that the neurons encode the current stimulus when the stimulus information is low and the difference image when the stimulus information is high.

## Methods

### Animals and initial surgery

All experimental procedures were approved by the Stockholm Regional Ethics Committee and were performed according to European Community guidelines for the care and use of animals in scientific experiments. Recordings were performed in 9 adult female ferrets. The ferrets were initially anesthetized with 15 mg•kg^−1^ ketamine hydrochloride (Ketalar, Pfitzer AB, Täby, Sweden) and 0.3 mg•kg^−1^ medetomidine hydrochloride (Domitor, Orion Pharma, Orion Corportion, Espoo, England) supplemented with 0.15 mg•kg^−1^ atropine (NM Pharma AB, Stockholm, Sweden). After initial anesthesia, the ferrets received a tracheotomy and were artificially ventilated (KTR-4 Hugo Sachs Elektronik, Harvard Apparatus GmbH, March Hugstetten, Germany) with 1∶1 N_2_O∶O_2_ and 1% Isoflurane (Abbott Scandinavia AB, Solna, Sweden). A craniotomy was made exposing the left hemisphere's visual areas 17, 18, 19 and 21 and was then covered with a chamber affixed to the skull with dental acrylic. Then the animal received an intravenous injection of 1.25 mg•kg^−1^ dexamethasone (Boehringer Ingelheim, Germany). To minimize eye drifts the animals were intravenously paralyzed with 0.6 mg kg^−1^ h^−1^ pancuronium bromide (Organon, Oss, The Netherlands). The left eye was occluded, and in the right eye the pupil was dilated with 1% atropine sulphate eye drops (Alcon, Alcon-Couvreur, Puurs, Belgium), the nictating membrane retracted with 10% Phenylephrine (Novopharma AG, Seinhausen, Germany) and the eye was then fitted with a zero power contact lens (Nordiska lins, Göteborg, Sweden). During surgery and recordings the body temperature, EKG and EEG were monitored and the expiratory CO_2_ was maintained between 3.3% and 4%.

### Visual stimuli

Visual stimuli were presented on a CRT monitor, subtending 22°×22° of visual angle at 57 cm. A Cambridge research systems video card was running the monitor with 800×600 pixels resolution and a vertical refresh rate of 120 Hz.

#### Visual stimuli for the decoding

**Figure 1:**
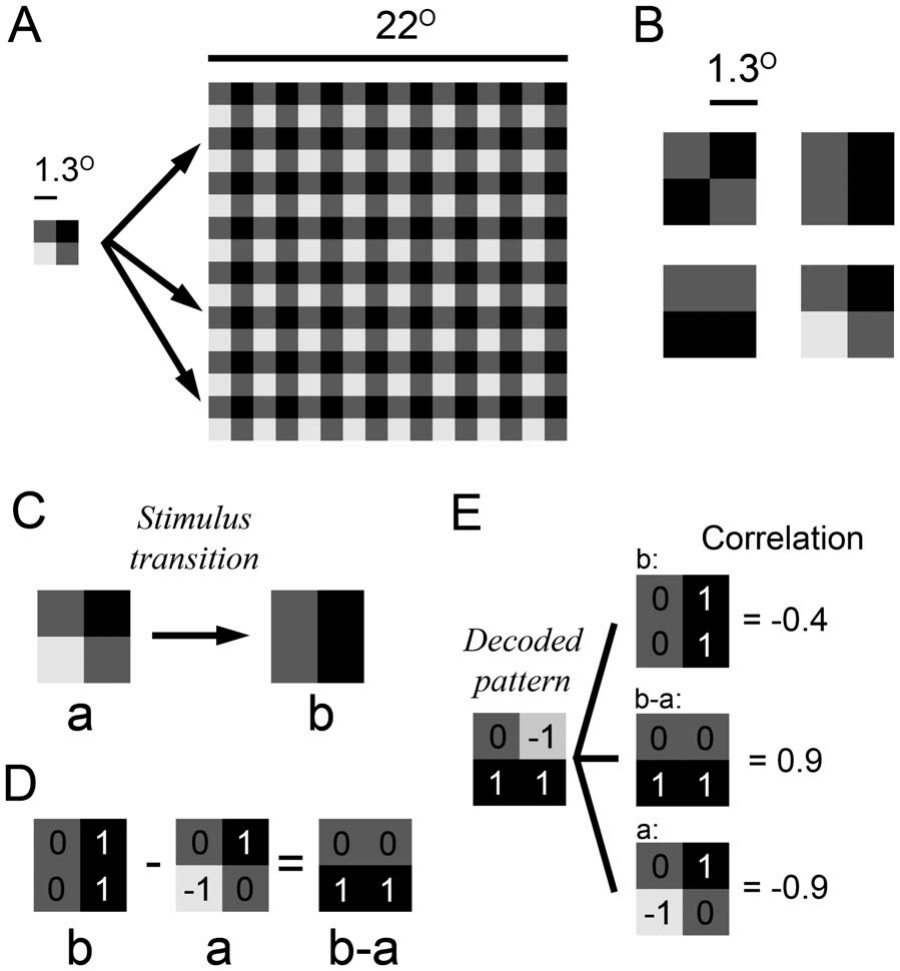
Visual stimuli used in this study. A: All stimulus patterns used in this study were covering 22×22° of the visual field. A luminance pattern consisting of 2×2 squares (each square was 1.3°) was repeated 8×8 times in order to cover the 22×22° screen area. B: Four examples of the 81 different patterns. C: An example of a stimulus transition. D: Calculating the difference pattern for the stimulus transition shown in C. E: The decoded pattern was correlated with the previous pattern, the current pattern and the difference pattern.

Each stimulus pattern was constructed by repeating a 2×2, (1.375°×1.375°) tiles ‘miniature image’ so as to cover the whole screen (Figure 1A). Each tile was assigned one homogeneous luminance value out of three possible, black (0.01 Cd/m^2^), gray (6 Cd/m^2^) or white (60 Cd/m^2^). The maximum number of possible patterns for 4 (2×2) tiles was 3^4^ = 81, which from now on will be referred to as P. A small number of pixels and pixel intensities is beneficial for decoding because we can map the cortical response to all possible pixel combinations.

The stimulus patterns were shown for 250 ms according to two different paradigms: a *relay paradigm* and *a dictionary paradigm*. The *dictionary paradigm* was used to extract the population code, i.e. the population activity in response to different isolated stimulus patterns. This dictionary was then used to decode the stimulus pattern from the population activity evoked by pattern transitions in the *relay paradigm* (the dictionary paradigm was used to decode the relay paradigm) or to decode the stimulus pattern from the population activity evoked by the *dictionary paradigm* (the dictionary paradigm was used to decode itself). In the *relay paradigm* a sequence of P patterns was shown in a randomly permutated order without any blank screen in between. In the *dictionary paradigm* the same P patterns were shown in a random sequence but with a homogenous gray screen lasting for 250 ms in between every pattern. The relay paradigm and dictionary paradigm were then interleaved and repeated 10 times. For each repetition the patterns were displayed in a new order (i.e. using a new permutation). The total number of permutations were therefore 20, i.e. 2 (for the two different paradigms)×10 (repetitions). Note that the same 20 permutations, and thus the same pattern sequence, were used for all penetrations (recording sessions). The recordings from all penetrations were pooled, except for the intra-areal analysis.

#### Visual stimuli for the orientation test

First, the orientation preference of each multiunit was examined by showing a gray background (20 Cd/m^2^) for 250 ms, followed by a stationary grating (5 and 35 Cd/m^2^) shown for 250ms at 16 different orientations in 22.5° steps, each repeated 10 times. To test the orientation encoding after a transition, the same grating as above was preceded by a 250 ms pattern (0, 10 and 60 Cd/m^2^, see Figure 1C) so as to produce the stimulus transition shown in Figure 1C. The orientation in the difference pattern is orthogonal to the orientation in the current stimulus pattern (see Figure 1D). The orientation in the difference pattern can be extracted as follows. Assume that the upper left, upper right, lower left and lower right luminance of the previous pattern is 0, 10, 10 and 60Cd/m^2^. The corresponding pixels of the current pattern are 5, 35, 5 and 35Cd/m^2^, i.e. a vertical line. The average luminance of the left two tiles (5 Cd/m^2^) will not change during the transition. The same holds for the right two tiles (35 Cd/m^2^). Therefore there is no luminance change along the orientation of the current stimulus pattern, i.e. vertically, and as a result this orientation will not evoke a response. On the other hand the luminance change will be orthogonal to that pattern, i.e. horizontally. The orientation in the difference pattern is therefore orthogonal to the orientation in the grating. To quantify the encoded orientation this transition was displayed at 16 different orientations in 22.5° steps (each repeated 10 times).

### Electrophysiological recording and data collection

Known cortical landmarks, such as the vascular pattern, suprasylvian sulcus and lateral sulcus, were used to guide the electrode to areas 17 and 18 [33]. The extracellular signal of single/multiple neurons was recorded with single shank 16-site laminar multi electrodes (3MΩ), with 100µm between two neighboring leads, spanning 1.5mm (a1x16-5mm100-177, Neuro Nexus Technologies, MI, USA). See Figure S1 for a sample trace. The electrode was always inserted perpendicularly to the cortical surface. A reference mark on the top of the electrode array enabled us to monitor the depth of the electrode beneath the cortical surface during the experiment. The electrode was positioned such that the top recording site was 100–200µm below the surface. The electrode was attached to a RA16AC head stage (Tucker-Davis Technologies) and the signal was pre-amplified using RA16PA Medusa Preamplifier (Tucker-Davis Technologies). The signal was amplified (gain 40K) and band pass filtered (100Hz–10kHz) using the RA16 Medusa Base station. Finally the data was captured and written to a hard-drive using CED power 1401 AD-converter (Cambridge Research Systems) and Spike 2 Software (Cambridge Research Systems). All subsequent analysis were done using Matlab R13 (The MathWorks, Natrick, Massachusetts). Extracellular amplitude peaks that crossed three times the standard deviation in the extracellular signal were defined as spikes. A recording site was considered significant if the average (across patterns and time) firing rate in the dictionary paradigm was larger than the mean plus two standard deviations of the firing rate between −100 ms and 0 ms before the stimulus presentation.

### Initial data treatment for the dictionary and the relay paradigm

All recording sites were used irrespective of whether they showed significant responses or not (95 out of 393 were significant). The results did not change significantly if only significant units were included.

In the dictionary paradigm one 250 ms peri stimulus time histogram (PSTH) was created for each pattern presentation period to see the pattern ON-response, and one 250ms PSTH for each blank period to see the pattern OFF-response. Time 0 denotes the time of the transition and is always at the beginning of the PSTH. The dictionary was created using the average firing rate during the first 90 ms (or 90–250ms, or 0–250ms) after pattern onset and across all 10 repetitions. The average firing rate of one pattern was assigned to the appropriate position, *RR_pn_*, in the matrix *RR*, where the row *p* stands for pattern index and column *n* stands for recording point.

In the relay paradigm a 250 ms PSTH was created for each pattern presentation period. The decoding was always done on the single trial population firing rate and not on the average population firing rate over many repetitions (as in the case of extracting the dictionary).

### Decoding

A new matrix R was created by normalizing each row in the matrix RR according to the following standard formula:
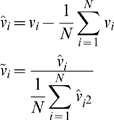
(1)This normalization enables us to calculate a correlation between the dictionary firing rate vector and relay firing rate vector by means of a multiplication and summation in equation 2 below (see discussion for a biological motivation).

**Figure 2:**
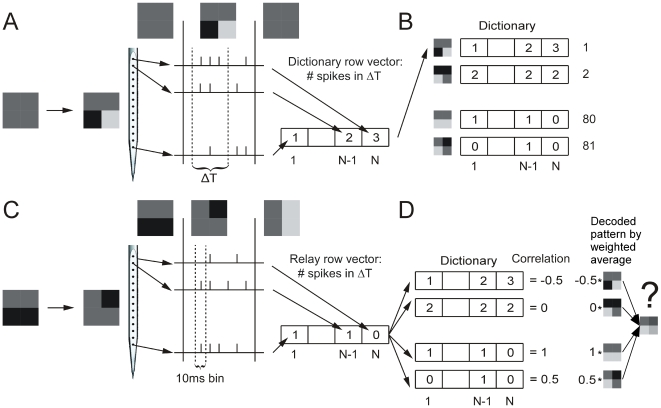
The decoding procedure. A: A pattern transition in the dictionary paradigm from a blank screen to a pattern. Multiunit activity was recorded with a single shank 16 leads electrode. The number of multiunit spikes was counted in a predefined time interval, ΔT, during the display of the pattern. In this study we used three different intervals, 0–250 ms, 0–90 ms and 90–250 ms. The number of multiunit spikes for each recording site was assigned to a vector (dictionary vector). This vector was associated to the presented stimulus pattern. B: Such a vector was then calculated for each of the 81 different patterns. C: A pattern transition in the relay paradigm (from one pattern to another pattern). The number of multiunit spikes was counted in 10 ms bins. The number of multiunit spikes for each recording site was assigned to a vector (relay vector). D: This relay vector was then correlated with each row vector in the dictionary. The resulting correlations were then used to weight the influence of the corresponding stimulus patterns. The weighted stimulus patterns were then averaged to create the decoded pattern.

The decoding was done on the instantaneous population firing rate in a temporal bin, rr, at one time point, t (see Figure 2C). The relay paradigm and the ON- and OFF-responses in dictionary paradigm were decoded using temporal bin sizes between 1 and 50 ms. The above defined normalization was done on rr so as to create r (see equation 1). r was then multiplied with the dictionary matrix R. The resulting vector, c, has P elements, one element for each pattern. Element *c_p_* tells how likely it was that the corresponding stimulus pattern, *p*, evoked the population firing rate *r*. More precisely element *c_p_* denotes the correlation between the population firing evoked by pattern p and the normalized instantaneous population firing r. A simple way to decode the population firing rate is to select the element in c with the largest correlation. The pattern corresponding to this value is the decoded pattern. This is the basic principle of the decoding method used in this paper.

This approach, however, will not include the information inherent in the correlations with the remaining P-1 patterns. A pattern that has a large correlation should be weighted stronger than a pattern that has a lower correlation. Therefore the decoded pattern was the correlation weighted average of all patterns. In order to create a weighted average of all patterns, the luminance contrast of each pattern was described by a vector with four elements. The value in each element was either 1 (black), 0 (gray) or −1 (white). Each pattern vector was then normalized (using equation 1), and organized into a matrix S with P rows and 4 columns. The luminance contrast value in each tile, d_l_, of the decoded pattern, d, was calculated as follows:
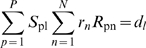
(2)The decoded pattern vector d was normalized in the same way as the stimulus pattern (see equation 1). For a particular time after a transition from pattern A to B (Figure 1C), the decoded pattern vector was correlated with the normalized stimulus pattern A, the normalized stimulus pattern B and the normalized difference (see below) between stimulus pattern B and A, B-A (Figure 1E). Finally the correlations from all pattern transitions (typically 810 transitions per animal) were averaged and the standard error was calculated. See Figure S2 for an illustrative example; see also Figure S3B for the results using this method, and Figure S3A and Figure S3C for a comparison of the results for different methods.

The difference pattern (B-A) was calculated by element wise subtraction of the non-normalized stimulus patterns, i.e. the resulting values could be −2, −1, 0, 1, and 2 (Figure 1D). After that the resulting pattern was normalized using equation 1. Since the decoded pattern was correlated with the pattern (A, B or B-A), the similarity was characterized in terms of spatial pattern and not average luminance level. That is, if one or both of the two correlated patterns were constant (no spatial contrast) the result of the correlation would be 0.

### Data treatment for the information estimation

The mutual information was calculated separately for each recording site and separately for the relay paradigm and the dictionary paradigm. Only recording sites that showed a significant response to the dictionary paradigm were included in the analysis. The information conveying unit was the number of spikes within a 10 ms window. The following analysis was done at time t after pattern onset. First we calculated the minimum and maximum number of spikes for all P patterns, i.e. for example 0–7 spikes. This interval was then divided into four evenly sized sub intervals, i.e. 0–1, 2–3, 4–5, 6–7 spikes. For each pattern a discrete probability distribution was created by assigning each of the 10 spike count values (each pattern has been repeated 10 times) to one of the four spike count intervals. Now all P patterns give P discrete probability distributions where all distributions share the same spike count intervals. The resulting two-dimensional probability distribution was defined as P(r, s), where r denotes the spike count interval and s denotes the stimulus pattern. The information at time t was calculated according to the following formula:
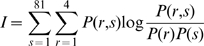
(3)To compensate for the firing rate bias, the information was also calculated for randomly permutated stimuli [34], [35]. The permutated information was subtracted from the un-permutated information. Information about the previous stimulus was calculated by counting spikes at time t+250 ms instead of at time t.

It should be noted that similar results were obtained with a more sophisticated probability estimation algorithm [36]. The probabilities were estimated with a neuronal network with a hidden layer with six units. The network was trained with error back propagation. The maximum number of iterations for convergence of the weights was set to 800. The momentum α was set to 0.5, and the weight update proportionality constant η was set to 0.005. The Matlab code and results can be retrieved upon request from the authors.

### Minimal model

The purpose of the minimal model is to approximate the image sequence encoded by the neurons in response to a stimulus image sequence. To this end we will predict the membrane potential for the ON-region of a neuron. The membrane potential (*u_ON_[t]*) is predicted by convolving the time course of the luminance (*sis[t]*) in the pixels covered by the ON-region with the temporal response function (*trp[t]*) of the average neuron.
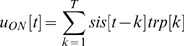
The temporal response function (*trp[t]*) is estimated with the reverse correlation described in Text S1 under *Mapping of the receptive field using reverse correlation*. For an OFF-region a luminance increase generates a membrane potential decrease, i.e. the temporal response function is inverted.
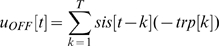
The encoded luminance (*eis[t]*) is then approximately represented by the membrane potential for the ON-region minus the membrane potential for the OFF-region.
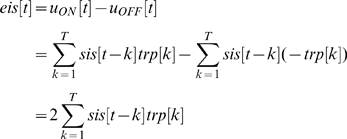
It is clear that this minimal model is a simplification. For example, there is no velocity representation (spatiotemporal interaction) or non-linear relation between membrane potential and firing rate. For the stationary patterns used in this paper, however, the model is able to qualitatively describe the results.

### Orientation preference

The firing rate for each orientation was averaged from 25 ms to 90ms after the onset of the grating. The orientation preference was approximated by the phase of a cosine function that was fitted to the resulting firing rate-orientation function. This calculation was done for the blank-grating and pattern-grating transitions (see *Visual stimuli for the orientation test* above). The difference in orientation preference between those two stimulus transitions was then calculated. A difference of 90° means that the orientation preference is dictated by the difference pattern rather than the currently shown stimulus pattern. A difference of 0° means that the orientation preference is dictated by the currently shown stimulus pattern.

## Results

In the first section we will correlate the decoded pattern with the previous, current and difference patterns. We will examine how these correlations evolve in time after a pattern transition. In the second section these correlations will be compared to the mutual information calculated for the same data set. Finally, in the third section we will examine whether the temporal modulation of the decoded pattern is intrinsic to every single unit or if it is the result of a combination of different neurons.

### The decoded pattern and its correlation with the previous, current and difference patterns

**Figure 3:**
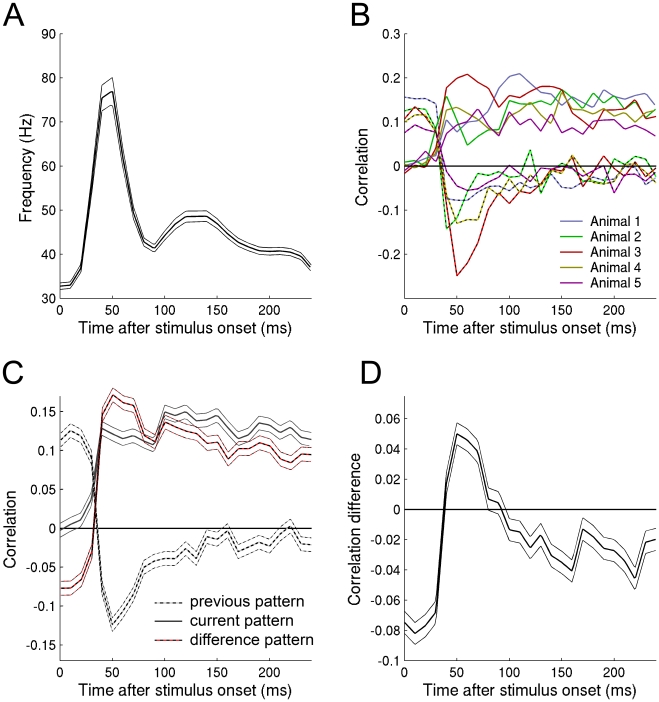
The decoded pattern changes with time. A: The average instantaneous firing rate after a stimulus transition. B: For each time point the decoded pattern was correlated with the current pattern (solid line) and with the previous pattern (dashed line). The average correlation was calculated across all 810 transitions per animal (see legend for color code). C: The correlation was averaged across all five animals and the standard error (thin lines) was calculated across all pattern presentations and animals. The standard error is the standard deviation divided by the square root of the number of data points. D: The correlation with current pattern was subtracted from the correlation with the difference pattern. Before 90 ms the decoded pattern is more correlated with the difference pattern than with the current pattern. After 90 ms the opposite is true.

We made 27 penetrations in five ferrets, where each penetration was performed using a single shank multi-electrode with 16 recording sites spanning all cortical layers, in area 17 and 18. In the relay paradigm we calculated a population rate vector each 10 ms (for other bin sizes see Figure S5). Given a population rate vector, we can use the dictionary (neuronal population code) to estimate what pattern was encoded (see methods). The resulting decoded pattern was correlated with the previous stimulus pattern and the current stimulus pattern. Unless otherwise stated we will use the average correlation across animals (Figure 3C).

Immediately after a stimulus transition the correlation with the current stimulus was 0 and the correlation with the previous pattern was 0.12±0.01 (p<10^−16^, n = 5*810, 2-sided paired t-test). This is expected since the neurons have not begun to respond to the new stimulus pattern. At 50 ms, when the average firing rate was maximal, the correlation with the current stimulus was 0.12 and the correlation with the previous stimulus was −0.12±0.01 (p<10^−15^, n = 5*810, 2-sided paired t-test). A correlation of 0.12 corresponds to 57% of the maximum correlation (see *Correlation upper bound* in Text S1), suggesting that neurons do not encode for either the current or the previous pattern.

The time course of the correlation with the previous and the current pattern can be described by the time course of the pattern encoded during the OFF- and the ON-responses respectively (see Text S1 section *Decoding ON- and OFF-responses*). Since a given spike will be influenced by the response to both the previous and the current pattern, the distinction of what is encoded (previous or current pattern) can only be done because the previous and the current pattern are known a priori. Therefore we want to examine what (single) pattern is encoded by the conglomerate of the ON and OFF responses. In other words what image is directly available in the neuronal firing? As the correlation with the current pattern is positive, +B, and the correlation with the previous pattern is negative, −A, the neurons seem to encode the difference between the current and the previous pattern, B-A. To test this we correlated the decoded pattern with the difference pattern (see methods for calculating the difference pattern) (Figure 3C). The correlation with the difference pattern at 50 ms, 0.17±0.01, was significantly larger than the correlation with the current pattern, 0.12±0.01 (p<10^−11^, n = 5*810, 2-sided paired t-test). As this value is relatively close to the maximal possible correlation (81% of the maximal correlation), this indicates that the difference pattern is the major component encoded by the neurons. After 90±15 ms (m±sd, n = 5) the correlation with the current pattern becomes larger than the correlation with the difference pattern. In Figure 3D we have subtracted the difference pattern correlation from the current pattern correlation. The current pattern correlation becomes significantly larger than the difference pattern correlation after 140 ms (p<0.05, n = 5*810, 2-sided paired t-test). From now on we will refer to the initial correlation with the difference pattern and the later correlation with the current pattern as the Difference-Current-characteristics.

### Mutual information

**Figure 4:**
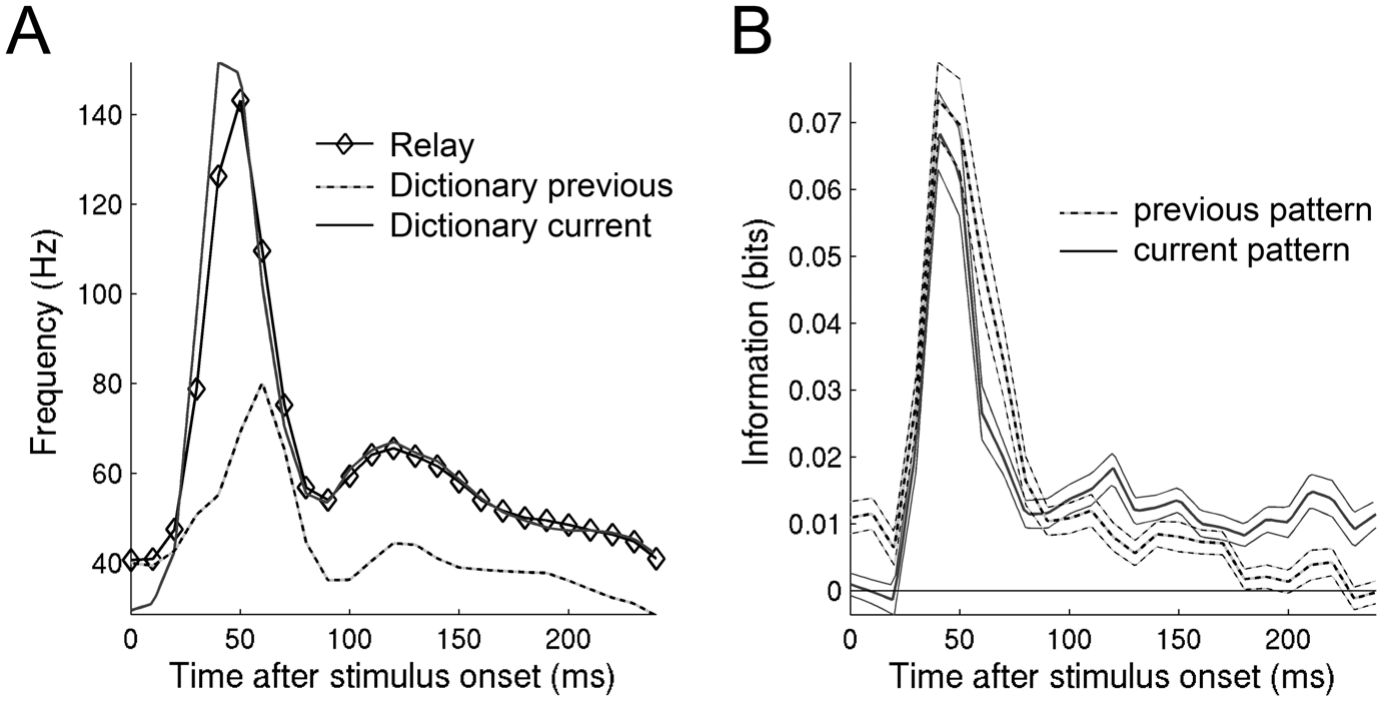
The difference image is encoded by the spikes that convey maximal information about current image. A: The average instantaneous firing rate for a pattern to pattern transition, a blank to pattern transition and a pattern to blank transition. B: The information about the current and previous stimulus was calculated in 10 ms bins. Initially the information about the previous stimulus is larger than the information about the current stimulus. After 100 ms the opposite is true.

If the difference pattern is encoded initially rather than the current pattern it would be counterintuitive if the neurons convey most information about the current pattern at this point. To examine this we calculated the average information for the relay paradigm from 95 significant multiunits (Figure 4B). Interestingly, the information about the current pattern is maximal when the correlation is larger with the difference pattern than with the current pattern.

The information can be large at 50ms because the signal to noise ratio is large at this time point. According to a Poisson process the signal to noise ratio is high because the absolute firing rate is high (Figure 4A). Therefore since the current pattern is a component of the difference pattern, and the fact that the difference pattern is encoded with a high signal to noise ratio it also means that the current pattern is encoded with a high signal to noise ratio (although not as high as the difference pattern).

The correspondence between the decoding and information becomes clearer as one compares the information between the current and the previous pattern (p<0.001, n = 95, 2-sided paired t-test). At 50 ms both previous and current patterns are equally represented. This is compatible with the encoding of the difference image since the difference image is related to both previous and current pattern. It is not until 180 ms that the information about the current pattern becomes larger than the previous pattern (p<0.0001, n = 95, 2-sided paired t-test). This is compatible with the encoding of the current pattern. Thus the Difference-Current-characteristics are also indirectly evident in the mutual information measure.

### Origin of the Difference-Current-characteristics

**Figure 5:**
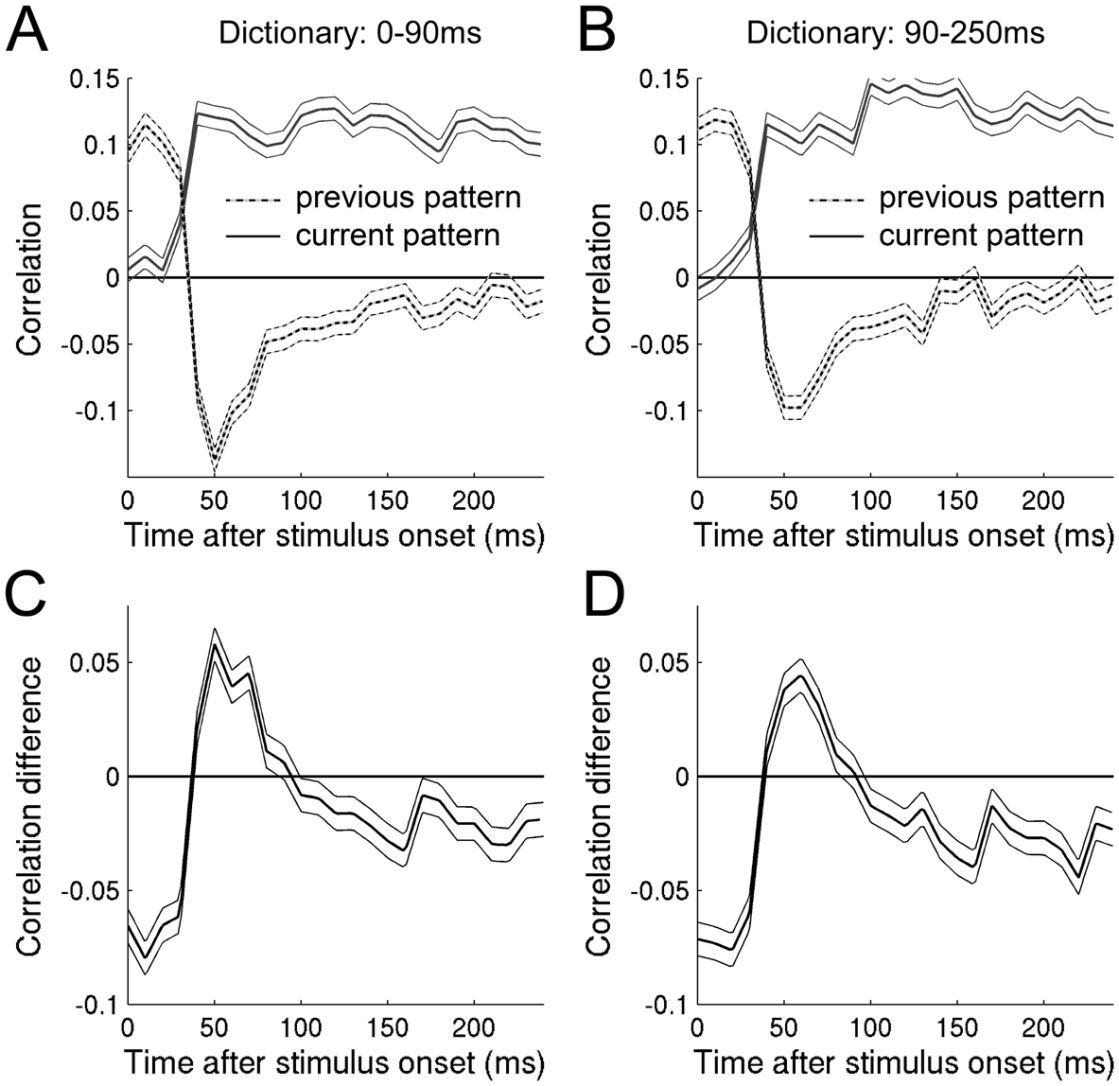
Dictionary estimated at two different time points. The decoding was done using two different firing rate codes. Either the code was the average firing rate between 0–90 ms after the blank to pattern transition (A), or the average firing rate between 90–250 ms after the blank to pattern transition (B). The difference between the correlation with the difference pattern and the current pattern was done for both codes (C, D). Note that both codes generated similar time courses.

Since the decoded pattern was different before and after 90 ms, one may suspect that the neuronal population code changes after 90 ms. In order to test this we extracted one dictionary from the average firing rate between 0 and 90 ms (ΔT = 0–90ms in Figure 2A), and another from the average firing rate between 90 and 250 ms (ΔT = 90–250ms in Figure 2B). The correlation curves for the two dictionaries do not differ significantly (dashed line in Figure 5A and B). This indicates that the neuronal population code before and after 90 ms is the same. Therefore it must be the same population of neurons that encode the difference pattern before 90 ms as those that encode the current pattern after 90 ms. Apart from a weaker difference coding in the infragranular layers, the Difference-Current-characteristics are not significantly dependent on cortical areas or layers (see Figure S7). More specifically this suggests that the Difference-Current-characteristics are intrinsic to the single unit, i.e the single unit transmits one message before 90 ms and another message after 90 ms (see discussion).

**Figure 6:**
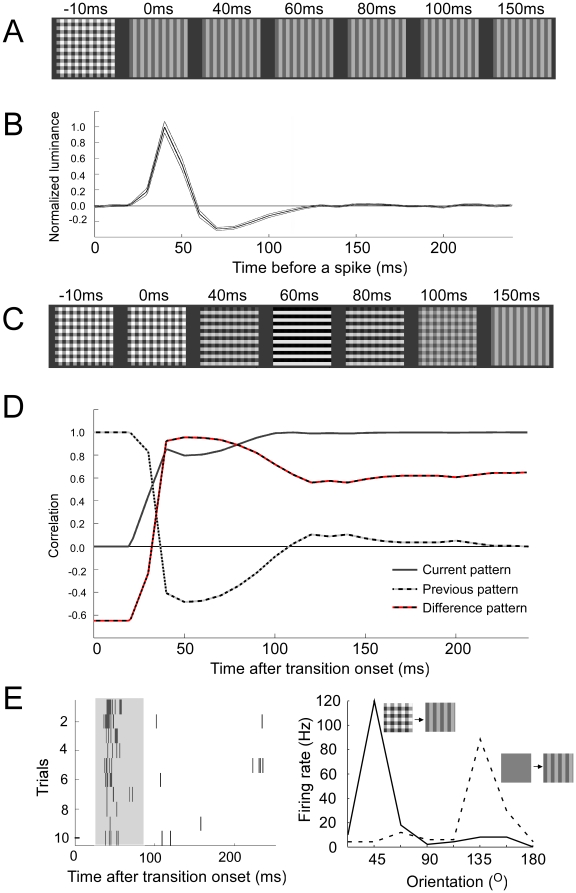
The Difference-Current-characteristics explained by the temporal response function of a single multiunit. A: Example of a stimulus pattern sequence that will be convolved by the temporal response function. B: The average temporal response function retrieved by reverse correlation of 8.3 ms frames of 16×16 tiles white noise stimulus. C: The decoded pattern sequence resulting from temporally convolving the stimulus in A with the average temporal response function in B. Note that the decoded pattern changes despite the constancy of the stimulus pattern; first the decoded pattern is horizontal lines and later it is vertical lines. D: The average correlation across all 81×81 possible stimulus pattern transitions. The correlation was done between the decoded pattern and current stimulus pattern (solid), previous stimulus pattern (dashed), and difference pattern (red dashed). Note the quallitative similarity with the population decoding result in Figure 3C. E: (right panel) Orientation preference of a square grating as it is preceded by a 250ms gray screen (dashed) or a 250ms previous pattern (solid). Note the 90° shift in orientation preference. E: (left panel) Spike raster plot for the pattern-grating transition at 45°. The shaded area denotes the time interval used to estimate the firing rate for the orientation preference in the right panel.

Can the linear spatiotemporal receptive field properties of a single neuron explain the Difference-Current-characteristics? To answer this we need to find the image pattern sequence encoded by the average single neuron. This encoded sequence can be approximated for any stimulus image sequence (for a natural scene movie see Movie S1) by convolving the luminance time course in every pixel by the temporal response function (see minimal model in the methods section). The temporal response function estimated by reverse correlation can be seen in Figure 6B (averaged across the receptive fields shown in Figure S4). Using this temporal response function, the pattern transition in Figure 6A, resulted in the encoded pattern sequence shown in Figure 6C. This model can now be used to estimate how each stimulus transition in the relay paradigm is encoded. For a given transition from pattern A to pattern B we correlated the decoded pattern with preceding pattern A, current pattern B, and difference pattern B-A. In Figure 6D one can see that the Difference-Current-characteristics are evident in this single cell model.

In Figure 6C we made the prediction that the message conveyed by the single unit may be at odds with the current stimulus pattern, i.e. the difference pattern has a horizontal orientation whereas the actual stimulus pattern has a vertical orientation. An example unit is shown in Figure 6D. The orientation preference of this unit was 135° (see Figure 6E). When the grating was preceded by a pattern the orientation preference was orthogonal to the true orientation. The average orientation shift across 17 orientation tuned units (cortical depth for those was 100, 200, 300, 300, 300, 400, 400, 500, 500, 700, 700, 800, 900, 1100, 1300, 1400 and 1500µm) in 4 animals was 89±15° (mean±sd, n = 17). Thus neurons are sensitive to the orientation in the difference pattern. Importantly the difference pattern orientation does not necessarily need to exist in the current pattern.

## Discussion

We introduce the apparent paradox that the response that conveys most stimulus-related information does not represent the stimulus itself. We find that rather than representing the stimulus, neurons initially encode a virtual stimulus that as a first approximation can be described as the difference between the current and the previous stimulus. The current stimulus becomes more exclusively or uniquely represented after 90 ms. We suggest that this representation change is due to a change in the transmitted message rather than a change in the neuronal code.

### Biological plausibility of the decoding algorithm

The decoding is done by reading the population activity pattern in the striate cortex in the same way as a simple cell is thought to be selective to the population activity pattern in the lateral geniculate nucleus. Since this population firing pattern readout is thought to be the general principle for the feedforward hierarchy in the visual system, this population pattern readout is biologically plausible [23]. We need, however, to motivate the additive and multiplicative normalizations done in equation 1. Cortical neurons are correlated in additive as well as multiplicative ways. Additive correlations may arise due to additive spontaneous activity [37]. Multiplicative correlations emerge as the population firing rate decreases during for example adaptation. Equation 1 could be implemented by an extra striate area in the same way as the early visual system performs luminance equalization (additive normalization) and contrast normalization (multiplicative normalization) of the retinal image. But instead of decorrelating activity across different photoreceptors in the retina, the extra striate area would decorrelate activity across neurons in the striate cortex.

The primary assumption for the method is that there is a monotonic relation between the firing rate and the luminance pattern. This assumption, while true for simple cells, is not true for complex cells. For a complex cell a certain increase in the firing rate can be the result of a luminance increase or decrease. Moreover a complex cell is not sensitive to where in its receptive field the luminance increase was. A firing rate increase is therefore insufficient to decode the spatial luminance contrast. The simple cell is therefore the major contributor to the decoding results presented in this study. It should, however, be noted that the orthogonal orientation coding in Figure 6E was evident in simple as well as complex cells.

### The neuronal mechanism underlying the Difference-Current-characteristics

What is the source of the temporal characteristics of the decoded pattern? Are the Difference-Current-characteristics a result of a mixture of different neurons? For example, could the difference-characteristic be explained by one population of cells at 50 ms, and the current-characteristic be explained by another population of neurons at 150 ms? Or does the single neuron change its representation as time unfolds? The latter possibility is more likely than the former. This is because the decoded pattern at 50 ms as well as at 200 ms was independent on whether the population rate code was taken from 50 ms or from 200 ms (Figure 5A and B). In theory, this could be explained by two different populations of neurons by chance in five out of 100 times assuming a significance level of p<0.05. It is, however, highly unlikely that such chance would produce a correlation with a p-value of less than 10^−9^ (When the decoding of 0–90ms is based on the dictionary from 90–250ms, Figure 5D). This suggests that the individual single unit first encodes the difference pattern and later encodes the current pattern.

Previous studies also suggest that the Difference-Current characteristics are intrinsic to the single units. As the average temporal response function in this study (Figure 6B) had a profile similar to the temporal response function from single cell studies using natural scenes for the awake monkey [8], the Difference-Current-characteristics could be explained by a single cell. Previous indirect information measures indicate that single cells convey information about the previous stimulus [38]. How much information the single neurons convey about the previous stimulus relative to the current stimulus, however, remains to be studied. Furthermore the single neuron conveys information about the previous luminance up to 500 ms after a luminance change [7]. In a recent single unit discrimination study, cells carried information about the previous stimulus up to 700 ms after the stimulus offset [27]. In contrast to our stimulus in which the local luminance changes in every pixel for the image transition, the long memory trace could be because the authors used letters A, B, C, D, and E, which don't overlap completely; when a new pattern is presented it means that the OFF-response for the previous pattern is, at some retinal locations, not being interrupted by the ON-response to the new pattern [39]. As a result some of the single unit activity may be the result of long lasting, 500–1000ms, spatial selective rebounding OFF-responses [25]. All in all this suggests that the Difference-Current-characteristics are intrinsic to the single unit.

What neuronal mechanisms could create the difference pattern? The difference pattern is encoded by the absolute firing rate (in contrast to the change in the firing rate). Therefore the absolute firing rate during the new stimulus must be proportional to the spatial contrast pattern of the new stimulus *relative* to the spatial contrast pattern of the previous stimulus. Thus the firing threshold must be set by the previous pattern. The firing in response to the previous stimulus may for example cause a delayed hyperpolarization. A crucial distinction is whether the resulting hyperpolarization affects the neuronal firing that originally caused it, i.e. feedback adaptation or inhibition, or whether the neuronal firing is unaffected by the resulting hyperpolarization, i.e. feedforward adaptation or inhibition. With feedback adaptation, the hyperpolarization cannot be arbitrarily strong because that would result in less firing and in turn in less hyperpolarization. This enables a switch from the difference to the current pattern. Feedback adaptation can therefore as a single mechanism explain the difference current characteristics. Feedforward adaptation on the other hand can have an arbitrarily strong hyperpolarization and can therefore completely eliminate the influence of the current stimulus. This is not compatible with the difference current characteristics, where after a while the neurons encode the current stimulus. Feedforward adaptation together with an additional sustained input, however, can explain the difference current characteristics. Two types of feedforward adaptation are synaptic depression and feedforward inhibition, i.e. an inhibitory neuron that receives the same input as the neuron it inhibits [40]. Four types of feedback adaptation are calcium dependent potassium channels, sodium channel inactivation, sodium gated potassium channels, and inactivation of calcium channels in the lateral geniculate nucleus [39], [41]. Our results suggest that the mechanism operates on the order of 100 ms. Therefore the most unlikely mechanism seems to be feedforward inhibition which generally is in the order of 10ms, which leaves the most likely mechanism to be a type of feedback inhibition.

### Short stimulus pattern durations and the dependency on inter-stimulus interval

What happens if the pattern duration is short relative to the time it takes for the neurons to adapt? This would make the neurons adapt not only to the previous stimulus, but also to the stimulus before that and so on. Therefore the neurons will not encode the difference between the current and previous stimulus, but rather the difference between the current and the average of all the previous stimuli. For a random sequence of short duration stimuli this means that the average previous stimulus becomes smoothed and more like a gray screen. The difference pattern will in this case be similar to the current pattern. This could explain the accurate orientation encoding in a recent study that uses a random sequence of 30ms gratings [42].

What pattern would the neurons encode if there was a temporal gap (gray screen) between the patterns? To examine this we first note that the Difference-Current-characteristics could be predicted from the blank-pattern and the pattern-blank transition (compare Figure 3C and Figure S6). This suggests that the correlation with the previous pattern is independent on the correlation with the current pattern. In the case of a temporal gap between the two patterns, we therefore predict that these two correlation time courses should be shifted relative to each other with a delay that corresponds to the duration of the gap.

### Difference image in previous studies

It has long been known that neurons respond to temporal changes and derivatives of the stimulus [10], [43]. In one study the encoding of the derivative of the stimulus was shown explicitly using a Gaussian derivative model [44]. The temporal sensitivity was modelled by the derivative of a symmetric Gaussian. As a result of the derivative, this model has a perfect asymmetry in the temporal domain, i.e. the positive side cancels the negative side. Therefore this model only encodes the difference image. It does not allow the representation to change from the difference image to the current image. The Difference-Current-characteristics is interesting since it is unclear how the readout from a neuron is done when that same neuron encodes the difference image at one time point and the current image at another time point.

The remaining of previous research related to our study can be divided into information and decoding studies. Information studies have focused on the amount of information the instantaneous firing rate conveys about the current stimulus [11], [30]–[32]. As the current stimulus, and not the previous one, was the main focus in these studies, each stimulus was preceded by a blank screen. To our knowledge there is only one study in which each stimulus was preceded by another stimulus [38]. In this study the information conveying unit was the cumulative firing rate (instead of the instantaneous firing rate used in our study). In their study the cumulative information continued to increase after stimulus offset. We believe that this can be explained by the difference image since the difference image contains both the previous and the current stimulus.

Decoding studies have primary focused on if it is possible to restore the stimulus image sequence from the neuronal response. In a previous study the population firing rate of a group of neurons in the thalamus was used to decode the retinal image in response to a movie of natural scenes [19]. The retinal image was accurately decoded, and no comments of eventual difference images were made. The decoding was made using a code that minimized the difference between the actual stimulus and the decoded stimulus [18], [45]. In short, they first estimated the temporal response function (see above). Since the temporal response function defines a linear transform from the stimulus to the firing pattern, the decoded stimulus was then retrieved by convolving the firing pattern with the inverse temporal response function. By doing so they assumed that the neurons used a temporal code (the temporal response function). As a result, the retinal image cannot be analyzed before the firing pattern has been convolved with the inverse temporal response function. In contrast, in our study we have used a neuronal population code (instantaneous rate code) that is thought to be used to perform analysis of the retinal image [13], [14], [20]–[24]. Given that this code conforms with image analysis computations, we test what image this code encodes. So the temporal code encodes the retinal image accurately, whereas the rate code conforms with existing mechanisms of image analysis. Presumably, the best code would be a code that conforms with image analysis computations *and* accurately encodes the retinal image.

### Future studies

The Difference-Current characteristics allow the experimenter to separate the image represented by the initial burst from the image represented by the later firing, i.e. the difference image can be independent of the current image (See Figure 6). The separation of the initial burst from the later firing may be interesting since the burst and the later firing are thought to play different roles. The initial burst is hypothesized to work as a wake-up call such that the brain can process later spikes [46], [47]. The initial burst could also have other functions. The maximum information rate contained in the initial burst indicates that it could be used to process the retinal image. For example the initial burst is thought to feed back from higher areas to primary visual cortex in order to convey information for figure-background segregation [48]–[50]. This hypothesis can be addressed by labeling the initial burst with image A, and labeling the later firing with image B (this means that the current image must be B and the previous image must be B-A). If the neurons rely on the initial burst for the figure background segregation, the information from higher areas contains figure-background information for image A rather than for the actual image B. This may be one way to quantify how the initial burst contribute to the processing of the retinal image, and how the initial burst is transformed as it is transmitted across cortical areas.

The difference image can be interpreted as the error between the current pattern and a constant prediction of the previous pattern, and can therefore be seen as the optimal input to cortical areas that can be intrinsically driven or self sustained. The network is free to do associations and sequence recall when the stimulus is constant and the difference image is zero, i.e. no error. On the other hand, when the stimulus changes the network becomes driven by the stimulus because the difference image signals an error.

**Table 1:** Correlations for each parameter combination used for optimization the SVM.

	C = 10	C = 3	C = 1	C = 0.3	C = 0.1	C = 0.03	C = 0.01
l qp false	*0.055*	*0.060*	*0.065*	*0.070*	*0.085*	*0.080*	*0.080*
l qp true	*0.015*	*0.020*	*0.020*	*0.020*	*0.010*	*0.010*	*0.020*
l ls false	*0.040*	*0.050*	*0.060*	*0.070*	*0.070*	*0.070*	*0.070*
l ls true	*0.010*	*0.010*	*0.010*	*0.020*	*0.020*	*0.020*	*0.020*
gauss qp false	*0.060*	*0.070*	*0.070*	*0.070*	*0.080*	*0.080*	*0.070*
gauss qp true	*0.020*	*0.020*	*0.020*	*0.010*	*0.010*	*0.010*	*0.010*

Output represents the value of the correlation for the current pattern at 150 ms. Best performance is underlined.

Kernel Function: **l** = linear, **gauss** = Gaussian Radial Basis Function kernel, sigma = 16 (optimal in a range of 1–30).

Method Used: **qp** = quadratic programming, **ls** = least-squares method.

Scaling of the data points before training: **true**, **false**.

Value of the box constraint for the soft margin: **C**.

**Table 2:** Correlations for each parameter combination used for optimization the neural network.

	[0]	[2]	[3]	[5]	[6]	[8]	[10]
Tansig Trainlm	*0.015*	*0.010*	*0.015*	*0.025*	*0.035*	*0.035*	*0.025*
Tansig Trainrp	*-*	*0.020*	*0.025*	*0.050*	*0.055*	*0.040*	*0.050*
tansig trainbfg	*−0.005*	*0.010*	*0.010*	*0.015*	*0.020*	*0.020*	*0.015*
tansig traingd	*∼0*	*∼0*	*∼0*	*∼0*	*∼0*	*∼0*	*∼0*
logsig trainlm	*∼0*	*0.020*	*0.020*	*0.035*	*0.050*	*0.050*	*0.040*
logsig trainrp	*-*	*0.020*	*0.020*	*0.035*	*0.050*	*0.040*	*0.040*
logsig trainbfg	*∼0*	*0.005*	*0.015*	*0.015*	*0.020*	*0.020*	*0.015*
logsig traingd	*∼0*	*∼0*	*∼0*	*∼0*	*∼0*	*∼0*	*∼0*
linear trainlm	*0.040*	*0.010*	*0.015*	*0.025*	*0.025*	*0.025*	*0.020*
linear trainrp	*-*	*0.005*	*0.015*	*0.015*	*0.020*	*0.025*	*0.020*
linear trainbfg	*0.035*	*0.015*	*0.025*	*0.040*	*0.045*	*0.045*	*0.035*
linear traingd	*0.005*	*0.005*	*0.005*	*0.010*	*0.015*	*0.015*	*0.015*

The output represents the value of the correlation for the current pattern at 150 ms. Best performance is underlined.

In the first row the number of units in the hidden layer is shown. 0 indicates no hidden unit and no hidden layer. More than one hidden layer (1–3) with number of units in each layer varying form (1–10) was tested but the performance was always worse than with a NN with 1 hidden layer and all remaining parameters the same.

Transfer Function: **tansig** = tangent sigmoid, **logsig** = log sigmoid, **linear** = linear transfer function.

Training method: **trainlm** = Levenberg-Marquardt backpropagation, **trainrp** = Resilient Backpropagation (does only work with hidden layers), **trainbfg** = Broyden–Fletcher–Goldfarb–Shanno quasi-Newton backpropagation, **traingd** = Gradient descent backpropagation.

## Supporting Information

Figure S1 An example of the extracellular signal recorded with the 16 channel single shank electrode. The shaded region indicates the 250ms stimulus time interval.
(1.84 MB TIF)

Figure S2 An 2 MUA example of the population decoding. A: The transition that will be studied (top). The 16×16 tile pattern is made by repeating a 2×2 tile image 8×8 times as indicated by the green grid (bottom). B: The target pattern (left) and its average (across all 8×8 regions) 2×2 tile image (right). The difference between the target pattern and the previous pattern and its average 2×2 tile image (bottom). C: The receptive fields of two MUAs (top) and the average (across all 8×8 regions) 2×2 tile image for the corresponding receptive field (bottom). D: Which of the two patterns displayed in B are most similar to the receptive field of multiunits MUA_1_ and MUA_2_ shown in C? The degree of similarity is extracted by multiplying the pattern with the receptive field. This is done for all 4 combinations; Target* MUA_1_, Difference* MUA_1_, Target* MUA_2_ and Difference* MUA_2_. The target pattern is preferred by MUA_2_ and the difference pattern is preferred by MUA_1_. E: The same conclusion as in D but on the basis of the firing rate of MUA_1_ and MUA_2_ in response to the target and difference patterns when the respective pattern is preceded by a gray screen, i.e., the dictionary paradigm. F: The firing rate for MUA_1_ and MUA_2_ in response to the pattern transition shown in A. G: The decoded pattern based on the firing rate in F, i.e., the relay paradigm.
(0.07 MB TIF)

Figure S3 Comparison of four different decoding techniques. A: The correlation weighted average (see methods). B: The decoded pattern is the pattern that corresponds to the maximal correlation (see Methods). C: Back propagation neural network with optimal parameters. See Table 2. D: Multi-Class, Support Vector Machine Based Decoding. See Table 1.
(1.88 MB TIF)

Figure S4 Supplementary figure 4. Receptive fields from all 5 animals. To the right of each receptive field we have for illustration purpose depicted the mapping of that receptive field to the 2×2 tiled pattern. This is done by averaging all 8×8 (denoted by the green lines) 2×2 tiles of the receptive field. This averaging is motivated by the fact that the 2×2 stimulus pattern is repeated over the stimulus monitor according to the same 8×8 grid.
(0.16 MB TIF)

Figure S5 Dependency of temporal bin size. A: The correlation between decoded pattern and the previous and the current pattern for different temporal bins, 2, 5, 10, 25, 50ms. 0–250ms was used for estimating the average firing rate for the dictionary. B: The correlation with the current pattern was calculated using the average firing rate in a 2, 5, 10, 50 and 125 ms interval centered at 196 ms after the pattern to pattern transition, and the code was the average firing rate in a 2, 5, 10, 50 and 125 ms interval at 196 ms after the blank to pattern transition.
(1.07 MB TIF)

Figure S6 The correlation with previous and current pattern is independent of pattern complexity. The decoding was done as a pattern was preceded by a blank screen (solid), and as a blank screen was preceded by a pattern (dashed).
(0.40 MB TIF)

Figure S7 The Difference-Current-characteristics is evident for each cortical depth and cortical position. A: The decoding was done for four different cortical depths. B: The decoding was done within each penetration. Only penetrations that generated significant correlations are shown (each animal has its own color). C: Four penetrations were done within the same animal. D: Those four penetrations indicated by four yellow points on the operative field picture.
(1.82 MB TIF)

Figure S8 The decoded pattern depends on the global luminance change. A: The luminance difference for all possible pattern transitions. The decoding was done for pattern transitions with luminance differences between 0–9 cd/m2 (B), 9–18 (C), 18–60 (D). Note that the correlation with the current pattern at 50 ms was decreased for large luminance differences (D).
(0.67 MB TIF)

Movie S1 Encoded image sequence in response to a clip from the original trailer of Australia™.
(1.81 MB MPG)

Text S1 (0.05 MB DOC)
