# Supplementary figures and images for: Relating Information, Encoding and Adaptation: Decoding the Population Firing Rate in Visual Areas 17/18 in Response to a Stimulus Transition
Source: PLoS One. 2010 Apr 27;5(4):e10327. doi: 10.1371/journal.pone.0010327 (PMC2860500; doi:10.1371/journal.pone.0010327)

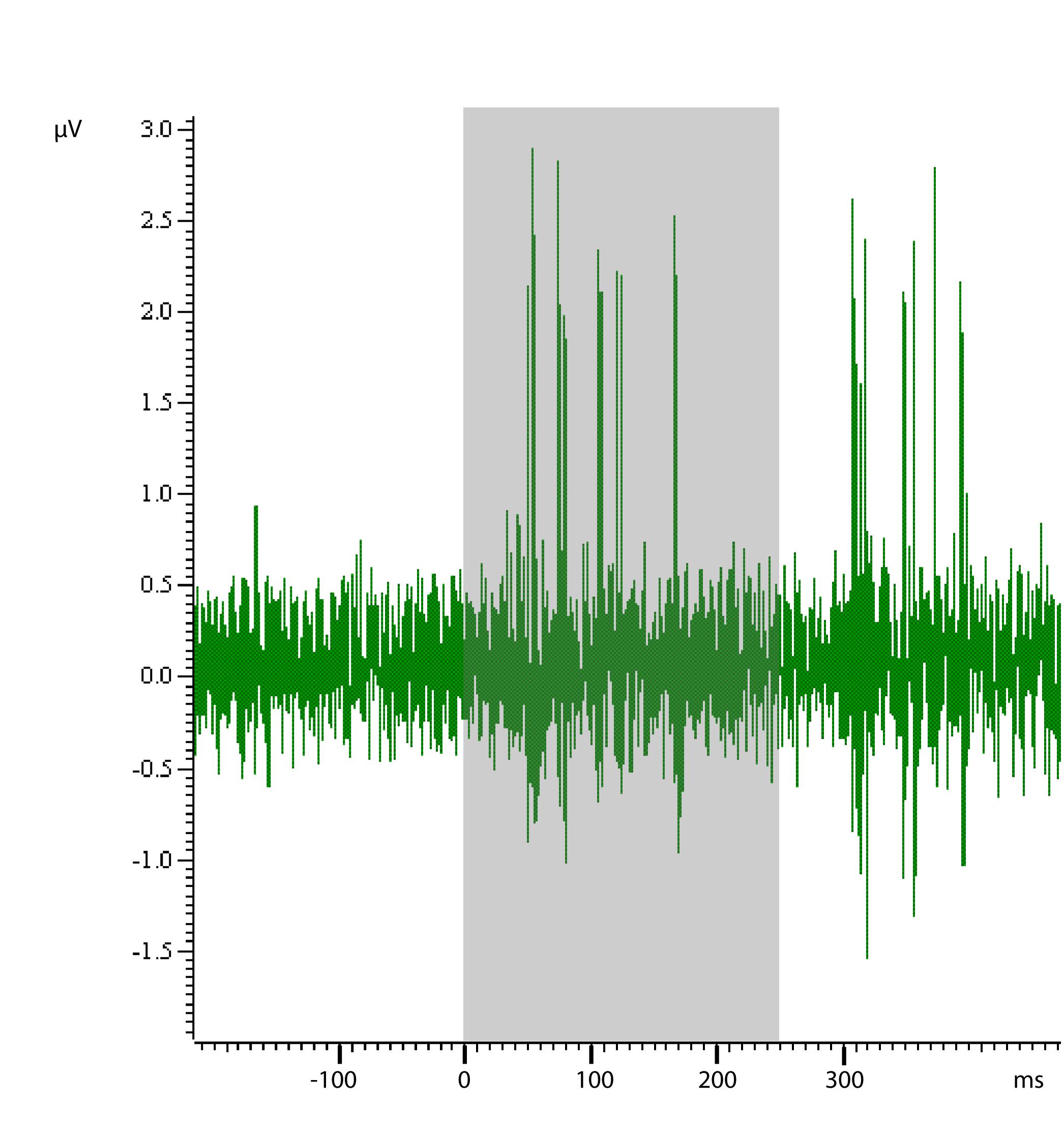

Supplement: Figure S1 — An example of the extracellular signal recorded with the 16 channel single shank electrode. The shaded region indicates the 250ms stimulus time interval. (1.84 MB TIF) [file pone.0010327.s001.tif]

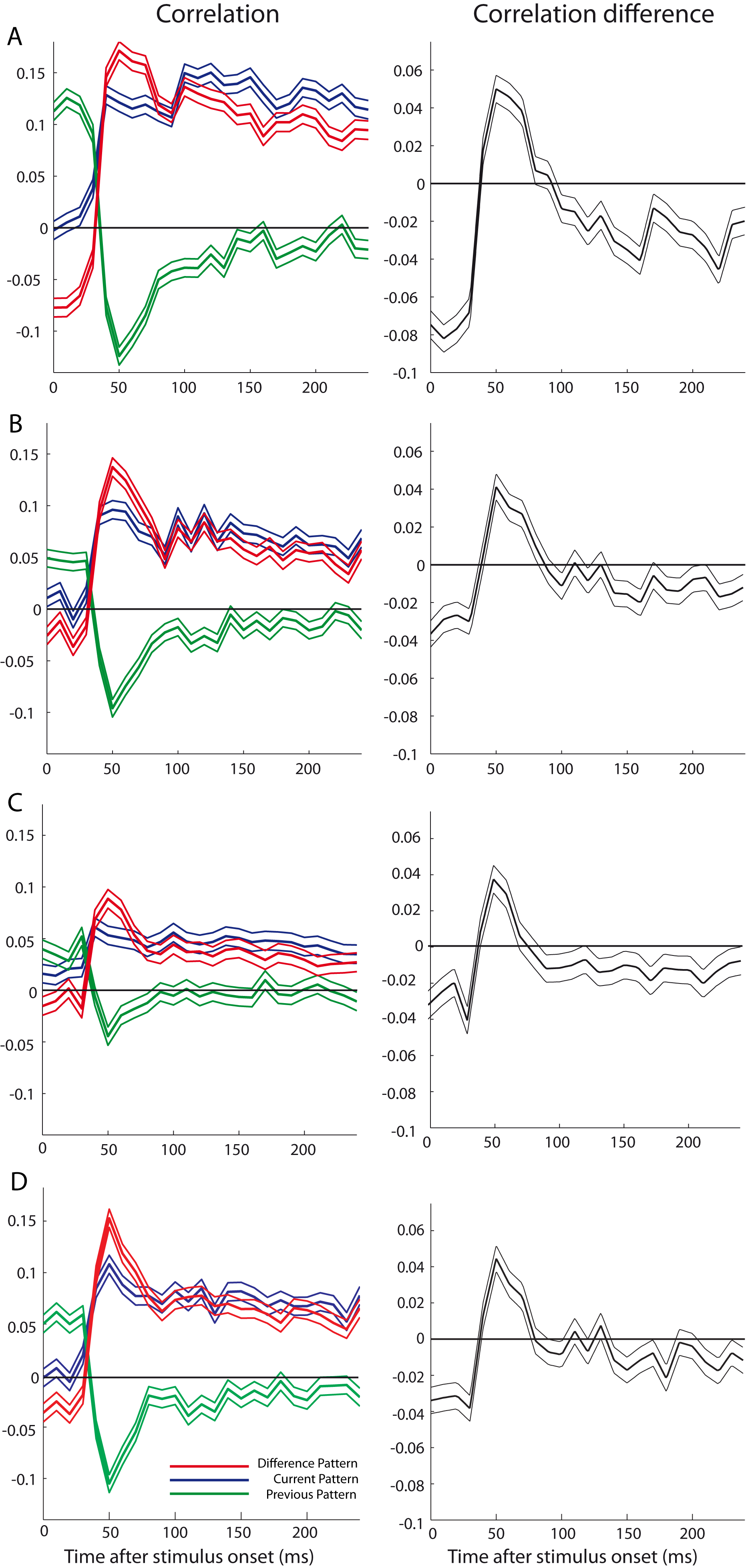

Supplement: Figure S3 — Comparison of four different decoding techniques. A: The correlation weighted average (see methods). B: The decoded pattern is the pattern that corresponds to the maximal correlation (see Methods). C: Back propagation neural network with optimal parameters. See Table 2. D: Multi-Class, Support Vector Machine Based Decoding. See Table 1. (1.88 MB TIF) [file pone.0010327.s003.tif]

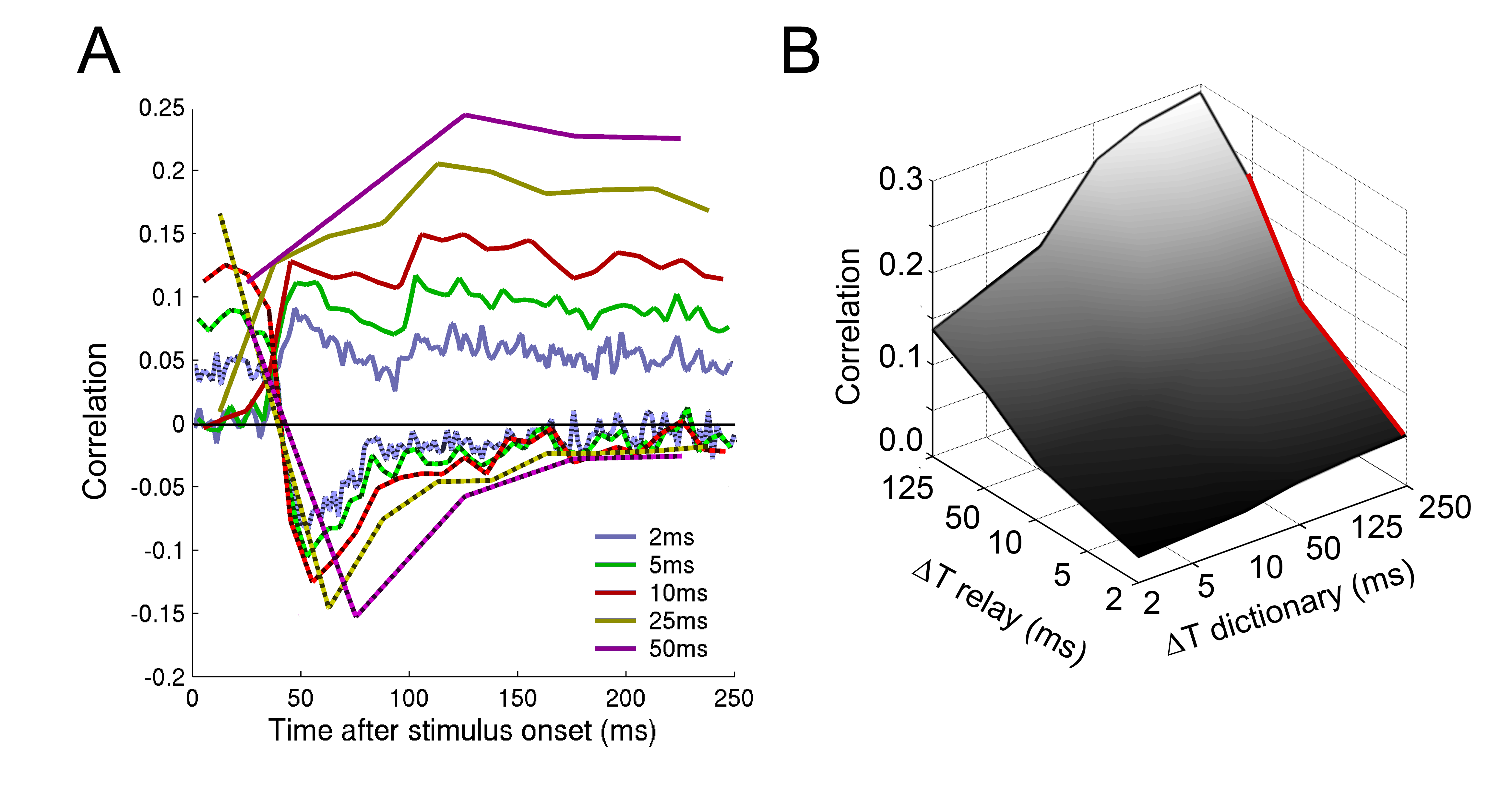

Supplement: Figure S5 — Dependency of temporal bin size. A: The correlation between decoded pattern and the previous and the current pattern for different temporal bins, 2, 5, 10, 25, 50ms. 0–250ms was used for estimating the average firing rate for the dictionary. B: The correlation with the current pattern was calculated using the average firing rate in a 2, 5, 10, 50 and 125 ms interval centered at 196 ms after the pattern to pattern transition, and the code was the average firing rate in a 2, 5, 10, 50 and 125 ms interval at 196 ms after the blank to pattern transition. (1.07 MB TIF) [file pone.0010327.s005.tif]

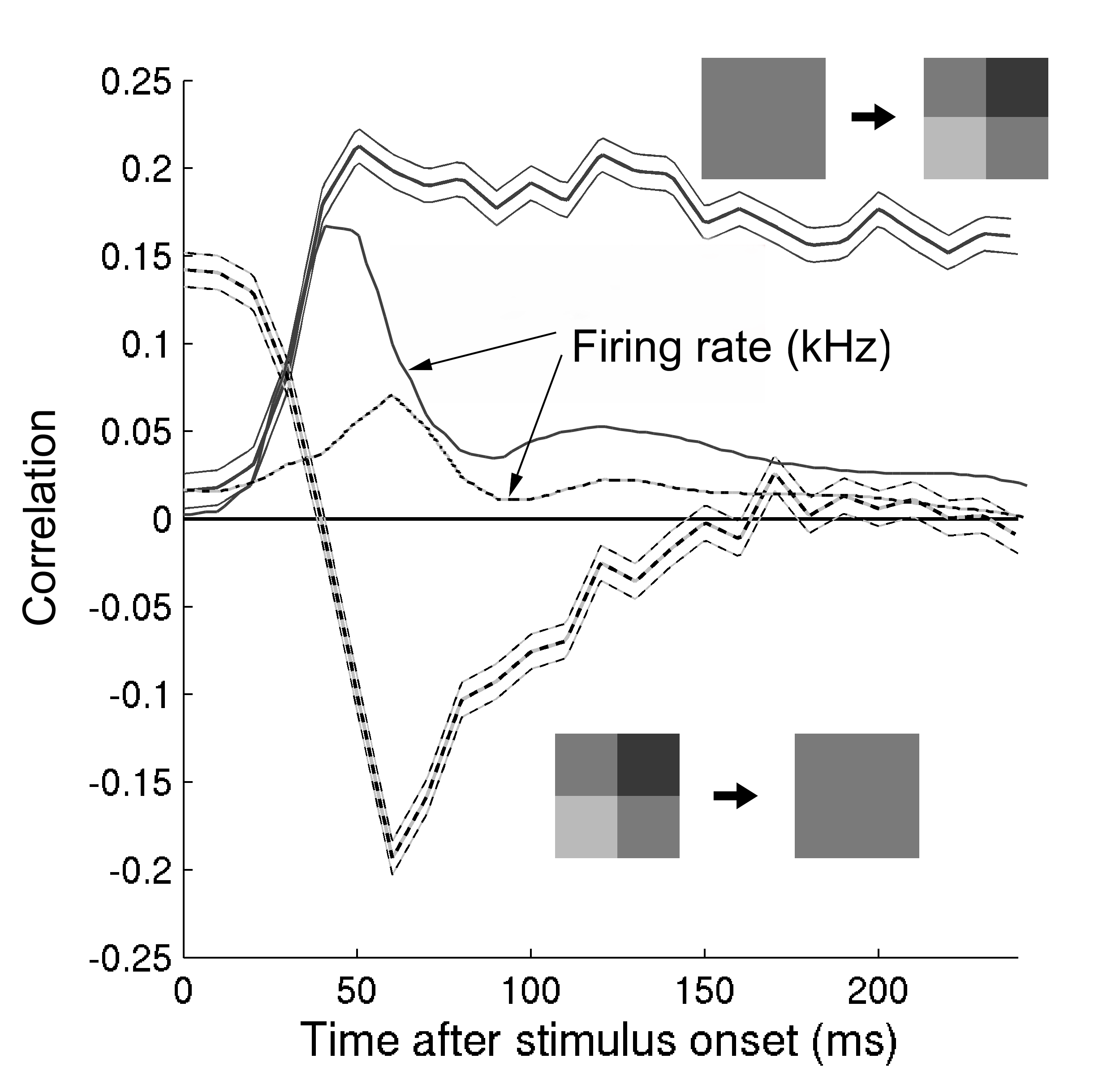

Supplement: Figure S6 — The correlation with previous and current pattern is independent of pattern complexity. The decoding was done as a pattern was preceded by a blank screen (solid), and as a blank screen was preceded by a pattern (dashed). (0.40 MB TIF) [file pone.0010327.s006.tif]

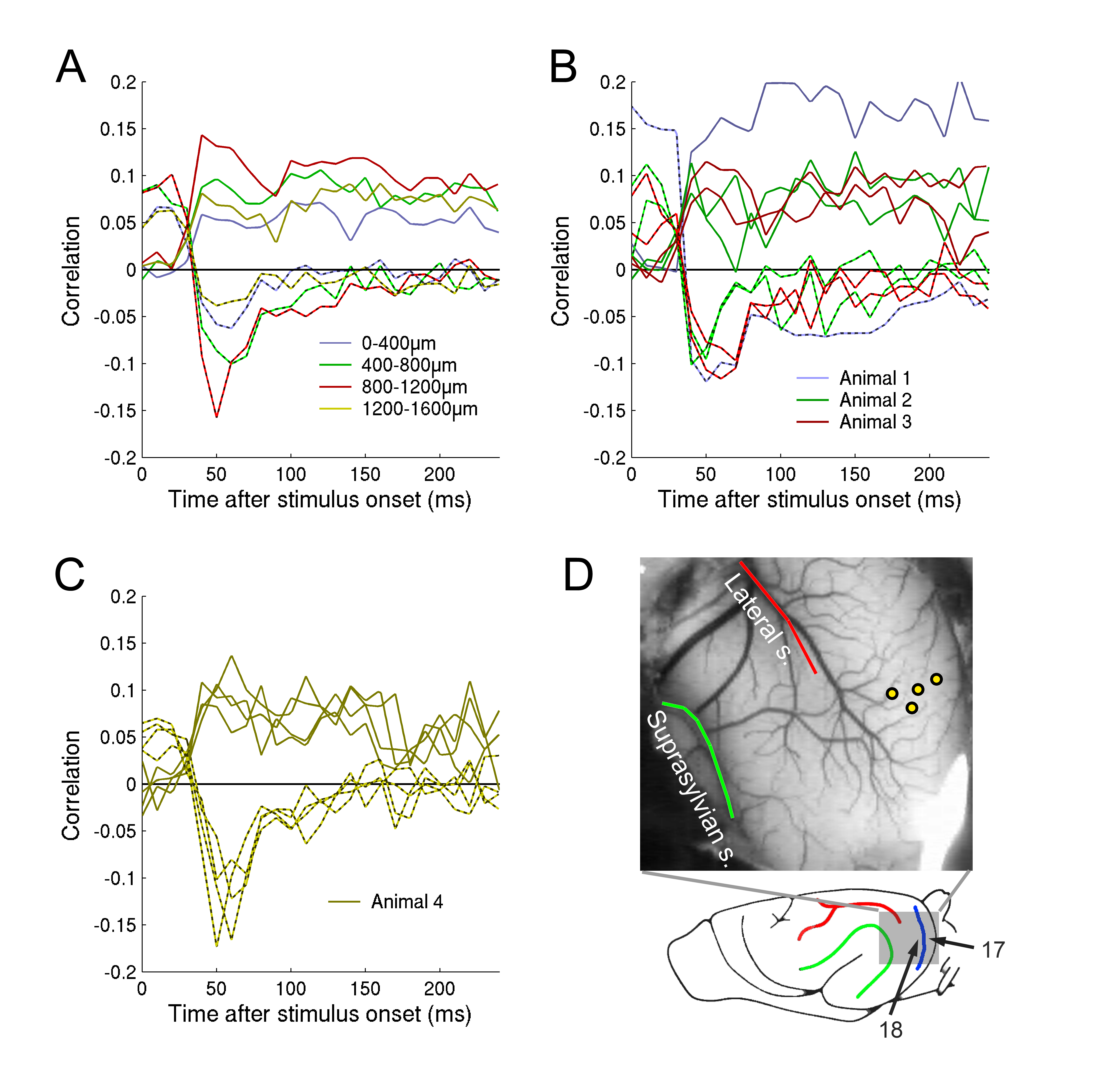

Supplement: Figure S7 — The Difference-Current-characteristics is evident for each cortical depth and cortical position. A: The decoding was done for four different cortical depths. B: The decoding was done within each penetration. Only penetrations that generated significant correlations are shown (each animal has its own color). C: Four penetrations were done within the same animal. D: Those four penetrations indicated by four yellow points on the operative field picture. (1.82 MB TIF) [file pone.0010327.s007.tif]

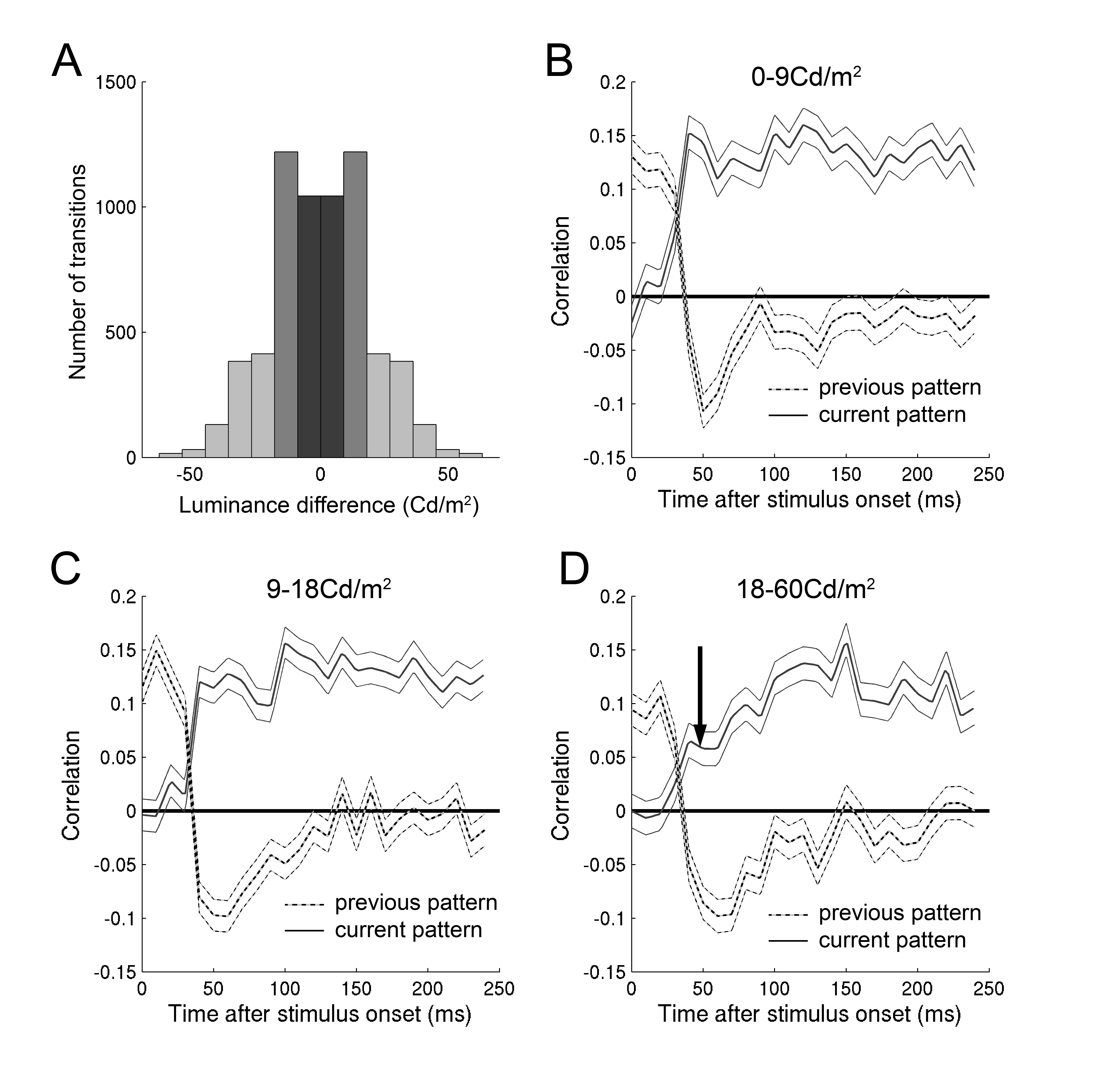

Supplement: Figure S8 — The decoded pattern depends on the global luminance change. A: The luminance difference for all possible pattern transitions. The decoding was done for pattern transitions with luminance differences between 0–9 cd/m2 (B), 9–18 (C), 18–60 (D). Note that the correlation with the current pattern at 50 ms was decreased for large luminance differences (D). (0.67 MB TIF) [file pone.0010327.s008.tif]
