# Supplementary material for: Relating Information, Encoding and Adaptation: Decoding the Population Firing Rate in Visual Areas 17/18 in Response to a Stimulus Transition
Source: PLoS One. 2010 Apr 27;5(4):e10327. doi: 10.1371/journal.pone.0010327 (PMC2860500; doi:10.1371/journal.pone.0010327)
Supplement: Text S1 — (0.05 MB DOC) [file pone.0010327.s010.doc]

**Supplementary information**

*Comparison of the different decoding methods*

The performance of four methods (weighted correlation, maximal correlation, support vector machine, artificial neural network) were compared by means of the steady state correlation at 150ms with the current pattern (Figure S3).

*Support Vector Machine (SVM)*

The algorithm was implemented in Matlab with the Bioinformatics Toolbox. We used a multiclass-SVM approach. We created 81 (the number of the different patterns) SVM classifiers; each trained to discriminate one pattern versus all the other patterns (One-Vs-All Classification). For every new input, we ran all the classifiers and chose the one that yielded the most positive output (f in [out,f] = svmdecision(Xnew,svm_struct)). For the optimization process we tried a number of parameters (table 1). Best performance was achieved with linear kernel and quadratic programming as the method to find the separating hyperplane. The value of the box constraint for the soft margin was 0.1 and auto-scale of the data points was disabled.

*Artificial Neural Network (NN)*

For the NN decoding method we used a feed-forward network with back-propagation training function. We tried different combinations of available parameters for a subset of our data (table 2). We found that the best performance was achieved with one hidden layer with 6 units and tangent sigmoid as transfer function. The training algorithm used was Resilient Backpropagation (trainrp). The network's performance was measured according to the mean of squared errors and training was stopped when the default tolerance was achieved or the maximum number of training epochs (100) was reached.

*Mapping of the receptive field using reverse correlation*

The same 16 x 16 tile grid used for the relay and dictionary paradigm was also used to map the spatiotemporal pattern of the receptive field [1]. For each frame refresh (120Hz) each tile was randomly set to either white or black. We pre-generated 32 768 frames. See Figure S4 for receptive field examples.

*Different bin sizes in the dictionary and relay paradigm*

The signal to noise ratio of the decoded pattern is expected to depend on the signal to noise ratio of the population rate. For example, is it possible to achieve a higher correlation than 0.12 (between decoded pattern and current stimulus pattern) by estimating the average firing rate more accurately? Since the accuracy of the average firing rate estimation is dependent on the number of spikes sampled, we calculated the average firing rate in intervals of different temporal lengths. In Figure S5A we have calculated the correlation for five different temporal bins, 2, 5, 10, 25 and 50ms in the relay paradigm. As the decoding depends on both the temporal bin in the relay paradigm and the dictionary paradigm we tested different temporal bin combinations for those two paradigms. For the dictionary population rate vector, the average firing rate was estimated using five intervals of 2, 5, 10, 50, 125 and 250ms duration, centered at 125 ms after stimulus pattern onset (that is, changing ∆T in figure 2A). For the relay population rate vector the average firing rate was estimated using five intervals of 2, 5, 10, 50 and 125 ms duration, centered at 196 ms after stimulus pattern transition. One of the resulting five dictionaries was then used to decode one of the resulting five population rate vectors at 196 ms. For each of those 25 combinations, the decoded pattern was correlated with the current stimulus pattern (Figure S5B). The correlation increases with increasing interval size and shows no sign of saturation at large interval lengths. Not surprisingly, the maximal correlation, 0.29, was achieved when both the dictionary and the relay paradigm were sampled at the largest interval.

*Correlation upper bound*

Here we will quantify the maximal possible correlation that is achievable with this decoding method. In information studies the stimulus information upper bound is usually estimated by preceding the stimulus by a blank screen [2-4]. Therefore by preceding a stimulus by a blank screen we expect the correlation between stimulus pattern and decoded pattern to be maximal. This was done with the data from the dictionary paradigm and the correlation at 50 ms was 0.21 (see Figure S6). Note that this correlation is the upper bound of the maximum correlation because decoding and code extraction (dictionary) were made on the same data. Therefore the maximum correlation is expected to be smaller than 0.21. This relatively low correlation can be attributed to the use of multiunits instead of single units, to the use of a relatively short bin size (10 ms), and to the fact that the decoding was made for single trial responses. Despite low correlation values, the consistency of the results was verified in two independent ways (orientation preference and minimal parameter-free model).

*Decoding ON- and OFF-responses*

In the correlation upper bound estimation we saw that the correlation time course with the current pattern is independent on whether the current pattern is preceded by another pattern or a blank screen. Here we show that the same is true for the previous pattern. That is, the correlation time course with the previous pattern is independent on whether the previous pattern is followed by another pattern or a blank screen (Figure S6). The correlation with the previous pattern reaches its maximal negativity 10 ms later when it was followed by a blank screen compared to when it was followed by a pattern. Furthermore the correlation with the previous pattern was significantly different from zero up to 130 ms, compared to 140 ms when the previous pattern was followed by another pattern. Based on these relatively small differences it seems that the correlation time courses of a pattern to pattern transition can be decomposed into the correlation time courses of the ON- and OFF-responses.

*Difference across cortical areas and layers*

Up to now the decoded pattern has been calculated using the activity from a mixture of neurons in areas 17 and 18 and all cortical layers. Here we will test how the Difference-Current-characteristics depend on the laminar location. The 16 leads of the electrode were divided into four depth regions with four leads in each region. Since the distance between two neighboring leads was 100 µm, each region spanned 400 µm, and the deepest region ended at 1600 µm below the cortical surface. The thickness of the gray matter in areas 17 and 18 of the ferret is between 1.2-1.3 mm in the perfused and stained brain [5], and between 1.4-1.5 mm in the freshly cut brain (unpublished observations). The average (across the animals that generate significant correlations) correlation in the four regions can be seen in Figure S7A. All regions generate a sustained positive correlation for the current pattern, and a negative correlation for the previous pattern at 50 ms. The clearest outliers (in terms of Difference-Current-characteristics) were the curves from the deepest region (corresponding to the infragranular layers), where the absolute amplitude of the negative correlation, -0.04, was smaller than the amplitude of the positive correlation, 0.08. This difference, however, was not significant (p > 0.05, n= 810*3). Absolute correlations are largest in the middle regions. This confirms that the method is most sensitive for simple cells and least sensitive for complex cells. This, however, does not exclude that complex cells encode the difference pattern (see figure 6E).

Here we will test if the Difference-Current-characteristics changes if we only record from one cortical area, area 17 and 18. To test this possibility the pattern was decoded using activity from a single penetration. A single penetration was used since the 16-lead shank was lowered orthogonally to the cortical surface and therefore the shank was unlikely to cross from one area to another. Trajectories from nine different penetrations in four different animals can be seen in Figure S7B and 7C (the other penetrations did not produce significant correlations). Each of these nine penetrations gave rise to the Difference-Current-characteristics. Four of these penetrations were done in one animal (Figure S7C), and their cortical location in relation to the cortical landmarks is depicted with yellow dots in Figure S7D. Since the largest distance between two penetrations was 1.2 mm (corresponding to the width of area 18), and since the line between these two penetrations was orthogonal to the presumed area border [5], it is unlikely that each penetration sampled from both area 17 and 18.

*Luminance dependency*

In a previous study it has been shown that a large change in the background luminance (8 cd/m^2^ or 18 cd/m^2^) can delay the information about a simultaneously presented stimulus [6]. In our study the average luminance varied across the different stimuli. Therefore we divided the pattern transitions into three luminance change groups, 0-9 cd/m^2^, 9-18 cd/m^2^ and 18-60 cd/m^2^ (Figure S8A). The correlation between decoded pattern and previous and current pattern for these three groups is shown in Figure S8B, 8C and 8D. The correlations for the two first groups do not differ significantly, but both of these groups are significantly different from the third group (p<0.01, n=5*810, 2-sided paired t-test). At 40 to 60 ms the correlation with the current pattern was approximately 0.12 for the first two groups (Figure S8B and C), whether it was 0.06 for the third group (Figure S8D). In contrast, the correlation with the previous pattern, between 40 and 60 ms, was similar for the three groups. Thus, it seems that a large luminance change results in a relatively stronger (negative) representation of the previous stimulus compared to the current stimulus.

**References**

1. Reid RC, Victor JD, Shapley RM (1997) The use of m-sequences in the analysis of visual neurons: linear receptive field properties. Vis Neurosci. pp. 1015-1027.

2. Heller J, Hertz JA, Kjaer TW, Richmond BJ (1995) Information flow and temporal coding in primate pattern vision. J Comput Neurosci. pp. 175-193.

3. Muller JR, Metha AB, Krauskopf J, Lennie P (2001) Information conveyed by onset transients in responses of striate cortical neurons. J Neurosci. pp. 6978-6990.

4. Richmond BJ, Optican LM, Spitzer H (1990) Temporal encoding of two-dimensional patterns by single units in primate primary visual cortex. I. Stimulus-response relations. J Neurophysiol. pp. 351-369.

5. Manger PR, Kiper D, Masiello I, Murillo L, Tettoni L, et al. (2002) The representation of the visual field in three extrastriate areas of the ferret (Mustela putorius) and the relationship of retinotopy and field boundaries to callosal connectivity. Cereb Cortex. pp. 423-437.

6. Huang X, Paradiso MA (2005) Background changes delay information represented in macaque V1 neurons. J Neurophysiol. pp. 4314-4330.
